# Laparoscopic splenectomy for solitary splenic metastasis in patients with previous open surgery—Case series

**DOI:** 10.1016/j.ijscr.2019.10.044

**Published:** 2019-10-28

**Authors:** Dobromir Dimitrov Dimitrov

**Affiliations:** a Department of Surgical Oncology, “G. Stranski” Hospital, Medical University, Pleven, Bulgaria; b Department of Surgical Propaedeutics, Faculty of Medicine, Medical University, Pleven, Bulgaria

**Keywords:** Laparoscopic splenectomy, Spleen metastasis, Case series, Case report

## Abstract

**Introduction:**

Solitary splenic metastases are a rare entity.

**Presentation of a case:**

We report two consecutive cases of solitary splenic metastases from ovarian and rectal cancer which were completely removed by laparoscopic splenectomy in patients with previous open surgery. The operative time was 165–200 min. The blood loss was 25–100 ml. There were no intra- and postoperative complications.

**Conclusion:**

Laparoscopic splenectomy for solitary splenic metastases seems safe and feasible with short recovery period even in patients with previous open surgery. More research in the area is needed.

## Highlights

• Two cases of laparoscopic splenectomy for solitary metastasis – patients after open surgery.
• Operative time was 165–200 min, patients were discharged on POD 3–4.
• Laparoscopic splenectomy is feasible after open surgery.

## 1 Introduction

Solitary splenic metastases are a rare entity. The most common malignant tumor of the spleen is lymphoma [1]. Others include primary splenic angiosarcoma or metastases [[2], [3], [4], [5], [6]]. Splenectomy and postoperative chemotherapy is the standard care for solitary splenic metastasis. Splenectomy could be performed open, hand-assisted laparoscopic, laparoscopic or robotic-assisted [[7], [8], [9]].

## 2 Methods

The research registry number in accordance with the declaration of Helsinki is researchregistry4988. Ethical approval has been obtained from the local ethics committee at Medical University – Pleven, Bulgaria. All patients signed informed consent. The study design is retrospective single-center case series conducted at Surgical Oncology Clinic, “G. Stranski” Hospital, Medical University, Pleven. The research has been reported in line with the PROCESS and SCARE 2018 criteria [10,11]. We report two consecutive cases of solitary splenic metastasis in patients with previous open surgery which were completely removed by laparoscopic splenectomy. Surgeries were performed by the same surgeon. Our team is experienced in laparoscopic and robotic colorectal surgery. This is our first experience with laparoscopic splenectomy.

## 3 Case presentation

The patient is given a pneumococcus vaccine 2–3 weeks prior to surgery. The patient is placed in right lateral decubitus position. Open technique for the insertion of the first trocar was used due to adhesions from previous surgery. Therefore, laparoscopic adhesiolysis was performed first and then lateral approach splenectomy by a 4-port technique. Selective ligation of the vessels in the hilum was done by hem-o-lok clips and the specimen was put in Endobag. Afterwards, a midline incision was made for specimen extraction. Patients were treated in our clinic within a period of a month.

### 3.1 Case 1

**Fig. 1:**
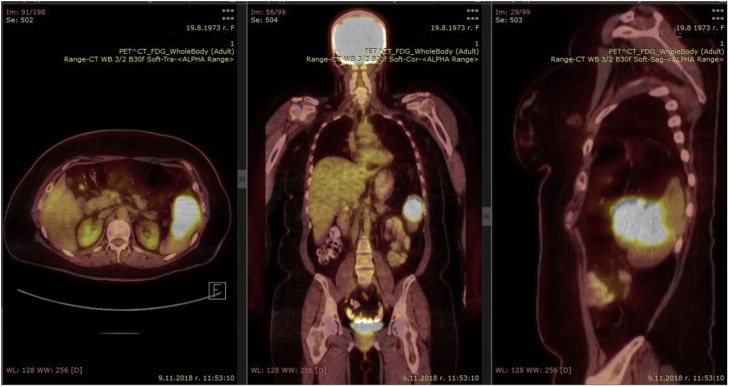
PET/CT of patient with spleen metastasis from ovarian cancer – case 1.

**Fig. 2:**
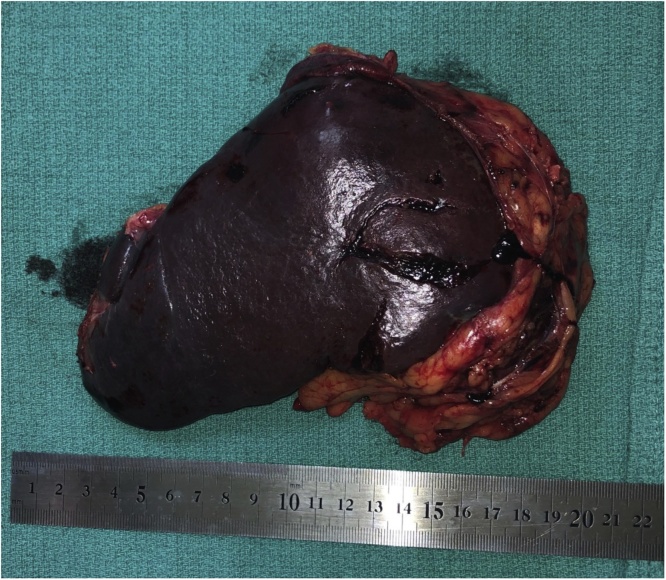
View of the specimen – case 1’.

A 45-year-old woman was found to have a solitary metastasis to the spleen from ovarian cancer. The patient was asymptomatic. BMI of the patient was 31.14 kg/m^2^, occupation – cashier in a supermarket, right-handed. Irregularly shaped lesion was found on a control CT in the lower pole of the spleen 18 months after the radical surgery for ovarian carcinoma with omentectomy (Stage IIIA) and adjuvant chemotherapy. Other medical, family and drug history was irrelevant. CEA and CA19-9 were in normal range. PET/CT was performed and no other lesions were found (Fig. 1). Laparoscopic splenectomy (Fig. 2) was carried out 4 weeks after the first splenic CT findings. The operative time was 200 min. The blood loss was 100 ml. There were no intra- and postoperative complications. Patient got up from bed and walked on POD 0. Diet was advanced on POD 1. The patient was discharged at POD 4. Histopathology and immunohistochemistry showed metastasis from ovarian cancer. On the 6-month follow-up on the whole-body CT there were no data for spread of the disease.

### 3.2 Case 2

**Fig. 3:**
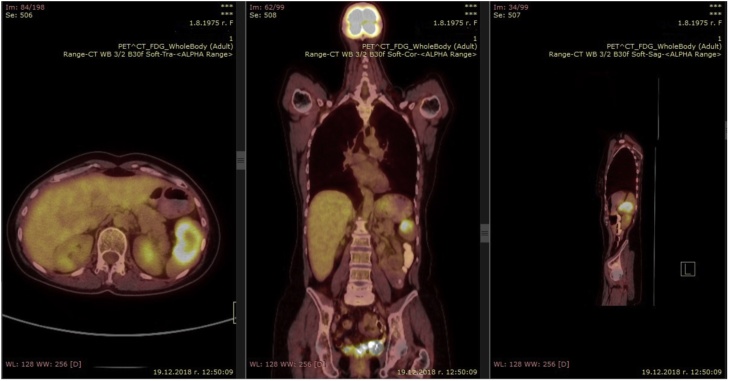
PET/CT of patient with spleen metastasis from rectal cancer – case 2.

**Fig. 4:**
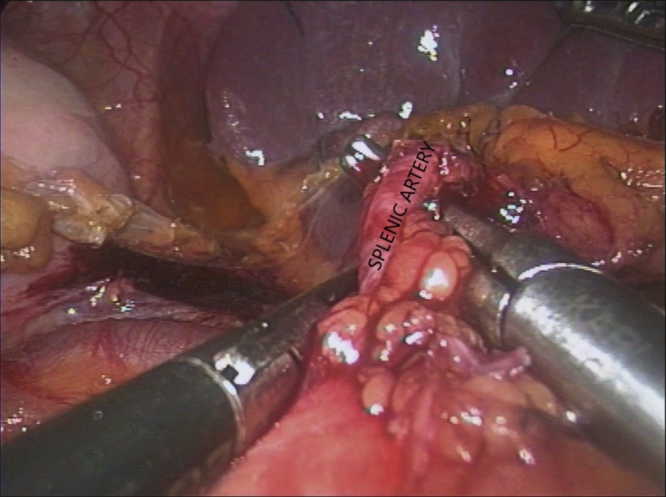
Intaoperative view of laparoscopic splenectomy – case 2.

A 43-year-old woman was found to have a solitary metastasis to the spleen. The patient was asymptomatic. BMI of the patient was 22.49 kg/m^2^, occupation – teacher, right-handed. A solitary lesion was found on a control PET/CT in the spleen 14 months after open total mesorectal excision with splenic flexure mobilization for rectal cancer (Stage IIIA) and adjuvant chemotherapy (Fig. 3). Open total hysterectomy was performed 27 months before the splenic lesion for an ovarian cancer stage I. Other medical, family and drug history was irrelevant. CEA and CA 19-9 were in normal range. Laparoscopic splenectomy (Fig. 4) was carried out 3 weeks after first splenic PET/CT findings. The operative time was 165 min. The blood loss was 25 ml. There were no intra- and postoperative complications. Patient got up from bed and walked on POD 0. Diet was advanced on POD 1. The patient was discharged at POD 3. Histopathology and immunohistochemistry showed metastasis from rectal cancer. On the 6-month follow-up on the whole-body CT there were no data for spread of the disease.

Both patients were satisfied with the shorter recovery period and less pain, especially compared to their previous experience with open surgery.

## 4 Discussion

Metastatic lesions of the spleen are a rare entity and may be discovered incidentally on imaging studies as in our cases. These are our first experience in laparoscopic splenectomy for solitary metastasis. Advances in diagnostic imaging techniques are mandatory for early diagnosis and therefore radical surgery. PET/CT is highly accurate in these cases differentiating malignant from benign lesions when assessing patients with a known primary malignant disease elsewhere [12]. Abi Saad et al. found only 26 cases reported with isolated splenic metastasis from colorectal carcinoma from 1966 to 2010 in their review [13]. Otrock et al. found only 16 cases reported with isolated splenic metastasis from ovarian carcinoma from 1955 to 2005 in their review [2]. Laparoscopic splenectomy was first used for benign hematologic diseases [14]. Nevertheless, laparoscopic splenectomy was found to be technically safe with several advantages over open splenectomy [15]. The adhesions in the area in both patients were no contraindication for laparoscopic surgery and there were no complications afterwards.

## 5 Conclusion

Advances in diagnostic imaging techniques are a prerequisite for an early diagnosis of the onset of metastatic diseases and thus the application of a minimally invasive approach in surgical treatment. Laparoscopic splenectomy for solitary splenic metastases seems safe and feasible with short recovery period even in patients with previous open surgery.

## Funding

No funding has been provided for this study.

## Ethical approval

Local ethics committee at Medical University – Pleven, Bulgaria has approved the study.

Number of approval: 605/04.07.2019.

## Consent

All patients signed informed consent.

## Author contribution

Dobromir Dimitrov – study concept and design, data collection, data analysis and interpretation, writing the paper.

## Registration of research studies

UIN: researchregistry4988.

## Guarantor

Dobromir Dimitrov.

## Provenance and peer review

Not commissioned, externally peer-reviewed.

## Declaration of Competing Interest

No relevant conflict of interest.
